# Fabricating High-Resolution and High-Dimensional Microneedle Mold through the Resolution Improvement of Stereolithography 3D Printing

**DOI:** 10.3390/pharmaceutics14040766

**Published:** 2022-03-31

**Authors:** Sangmin Choo, SungGiu Jin, JaeHwan Jung

**Affiliations:** Department of Pharmaceutical Engineering, Dankook University, Cheonan 31116, Korea; sangminchoo96@gmail.com

**Keywords:** microneedles, 3D printing, stereolithography 3D printing, transdermal drug delivery, printing angle, printing resolution

## Abstract

Microneedles are transdermal drug delivery tools that can be fabricated simply, economically, and rapidly using SLA 3D printing. However, SLA 3D printing has a limitation in that the resolution is slightly lowered when the microneedle is precisely printed. To solve this issue, we optimized the SLA 3D printing conditions such as printing angle, needle height, aspect ratio, and spacing between the microneedles for high-resolution microneedle fabrication. The sharpest microneedle tip was obtained when the printing angle was adjusted to 60° in both the x and y axes. The aspect ratio and the spacing between the microneedles did not affect the output of the sharp tip. Under optimal conditions, the microneedles with 1180 ± 20 µm height, 490 ± 20 µm base, and 30.2 ± 3.4 µm tip diameter were obtained. The dissolving microneedle patch, prepared using the 3D printed microneedle as a mold, penetrated the porcine skin ex vivo. When the printing angle was 60° in the x and y axes, the area of the single stacking layer, including the microneedle tip, increased, and thus the sharp tip could be printed. A high-dimensional, side-notched arrowhead (SNA) microneedle was fabricated by applying the SLA 3D printing condition. Moreover, a letter-type microneedle patch was fabricated using the customized characteristics of 3D printing. Consequently, high-resolution and high-dimensional microneedles were successfully fabricated by adjusting the printing angle using a general SLA 3D printer, and this technology will be applied to the manufacture of drug delivery tools and various microstructures.

## 1. Introduction

The transdermal drug delivery market is estimated to grow to approximately $95.77 billion by 2025 [1]. Microneedles, which compensate for oral administration and subcutaneous injection limitations, are attracting attention as an ideal transdermal drug delivery tool [2]. A microneedle 100 to 1500 µm in length can penetrate the stratum corneum without pain [3,4]. Moreover, since the drug is administered through the skin, the drug, which is difficult to deliver through the oral route of administration, can be efficiently delivered through the skin by avoiding the liver first-pass effect [5,6]. In general, a microneedle is fabricated precisely with resolutions of hundreds of nanometers by microelectromechanical system (MEMS) technology, including chemical wet etching, ion etching, and drawing lithography [7,8,9]. However, MEMS technology requires expensive equipment and has high maintenance costs, and the manufacturing method is complicated [10]. Therefore, it is essential to develop a technology that can economically and precisely manufacture microneedles, thereby lowering the entry barrier for microneedle research and revitalizing the development of microneedle pharmaceuticals and cosmetics.

3D printing, also called additive manufacturing, is a rapid and simple method to print three-dimensional objects economically. Furthermore, using 3D printing, the design of the printed product can be easily modified and supplemented through the CAD program. Thus, 3D printing is being applied to various industrial fields, including microneedle fabrication [11,12]. 

Microneedle fabrication methods using various 3D printing techniques such as Fused Deposition Modeling (FDM), Stereolithography (SLA), Digital Light Processing (DLP), and Two-Photon Polymerization (2PP) have been reported [13,14,15,16]. FDM is a cost-effective 3D printing method that melts and extrudes a thermoplastic material such as polylactic acid at a high temperature but has the disadvantage of low printing resolution [17]. SLA, DLP, and 2PP are photopolymerization-based technologies manufactured by irradiating and curing UV-curable resin with UV and have high resolution [18]. Since 2PP has a resolution of several hundred nm, it is suitable for manufacturing sophisticated and complex structures. However, it has a slow manufacturing speed and requires expensive equipment [19]. Therefore, research on microneedle fabrication has been conducted using SLA and DLP types of 3D printers that are economical and rapid to print, though the resolution is somewhat lower (Table 1).

Economidou et al. developed the pyramid and spear-shaped microneedles for insulin delivery using an SLA-type 3D printer. The insulin formulation was coated on the 3D-printed microneedle for transdermal delivery. In an in vivo animal experiment, the insulin-coated microneedle lowered the glucose level within 60 min and showed a faster and more sustained insulin action than a subcutaneous injection, but the resolution of the microneedle tips was low [14]. Krieger et al. manufactured a microneedle mold with a high-resolution tip using an SLA-type 3D printer, but there was a limitation that an additional process was required to implement a microneedle mold of an appropriate length [20]. Therefore, to overcome the limitations of the existing 3D printing-based microneedle fabrication, it is necessary to develop a 3D printing technology that can produce high-resolution microneedles in one step, rapidly, economically, and without additional processes.

**Table 1:** The advantages and disadvantages of various microneedle manufacturing methods.

3D Printing Method	Advantages	Disadvantages	Refs.
FDM	Cost effective	Low printing resolution	[17]
2PP	High printing resolution (hundreds of nm)	Expensive equipment Slow printing speed	[19]
SLA (Print and Fill)	Cost effective High printing resolution (tens of µm)	Requiring additional process	[20]

In this study, we demonstrated a simple printing method manufacturing high-resolution microneedles using an SLA 3D printer. 3D printing conditions were optimized by controlling the printing angle, microneedle height, aspect ratio, and spacing between microneedles as variables to fabricate high-resolution microneedles for transdermal drug delivery. The dissolving microneedle patch (DMP) was fabricated using the printed microneedle as a mold, and the mechanical properties and skin penetration potential of DMP were confirmed. By analyzing the 3D printing conditions of the optimized microneedle, a study was also conducted to produce a high-dimensional microneedle. Furthermore, using the characteristics of 3D printing, a customized microneedle patch in the shape of a letter was fabricated, and various application possibilities of the microneedle were studied.

## 2. Materials and Methods

### 2.1. Materials

The 3D printed microneedle arrays were fabricated using an SLA 3D printer (Form 3, Formlabs Inc., Somerville, MA, USA), UV-curable resin (Clear Resin V4, Formlabs Inc., Somerville, MA, USA), washer (Form Wash, Formlabs Inc., Somerville, MA, USA) and UV chamber (Form Cure, Formlabs Inc., Somerville, MA, USA). Sodium carboxymethylcellulose (CMC-Na) and sulforhodamine B (SRB) were purchased from Sigma-Aldrich (St. Louis, MO, USA). Sucrose was purchased from Millipore (Burlington, MA, USA). Polyvinylpyrrolidone K-30 (PVP K-30), and gentian violet solution was purchased from SAMCHUN (Pyeongtaek-si, Gyeonggi-do, Korea).

### 2.2. Design and Fabrication of Microneedles Using 3D Printing

The 3D printed microneedle arrays were created by CAD software (3DS MAX, USA). All designs were converted to STL files and uploaded to the slicing software (Preform, Formlabs Inc., Somerville, MA, USA). The layer thickness was set to 25 μm to increase the resolution [20]. The 3D printed microneedle arrays were washed for 20 min using Form wash and then cured at 60 °C for 1 h using Form Cure. To analyze the factors affecting the 3D printing, the printing angle was adjusted from 0, to 45, to 60°; the microneedle height was adjusted from 600 to 2000 μm with 200 μm intervals; and the intervals between the needles were adjusted from 250, to 500, to 1000 μm. The 3D printed microneedle arrays for the dissolving microneedle patch (DMP) were designed as 7 × 7 arrays with 1300 μm height, 500 μm base, and 1000 μm needle-to-needle distance. A stereomicroscope (SZ61 TR, OLYMPUS Ltd., Tokyo, Japan) and scanning electron microscopy (SEM; SU8230, Hitachi, Tokyo, Japan) were used to observe the 3D printed microneedle arrays.

### 2.3. Fabrication of the Dissolving Microneedle Patch (DMP)

The mold for DMPs were prepared by pouring polydimethylsiloxane (PDMS; Sylgard^®^ 184, Dow Corning, Midland, MI, USA) with the ratio of 10:1 into the 3D printed microneedle array. The PDMS-casted 3D printed microneedle was degassed in room temperature for 30 min and cured in the oven at 85 °C for 1 h. DMPs were fabricated by solvent casting method (Figure S1). First cast solution containing SRB as model drug was formulated by dissolving 2% (*w*/*v*) CMC-Na and 15% (*w*/*v*) sucrose in deionized (DI) water. Second cast formulation for forming the backing layer was prepared by dissolving 40% (*w*/*w*) PVP K-30 in DI water. A vacuum pump (MPC601T, Welch, Fürstenfeldbruck, Germany) was used to fabricate DMPs at −1000 mbar, and 100 μL of the 1st cast solution was poured onto the PDMS mold and vacuumed for 1 h. After removing the excess solution, the PDMS mold was dried for 1 h. Subsequently, 270 μL of the 2nd cast solution was poured onto the PDMS mold and vacuumed for 1 h. Then, the DMPs were dried overnight and peeled off from the PDMS mold. The prepared DMPs were stored in the desiccator for 24 h. A morphology of DMPs was observed using a stereomicroscope.

### 2.4. Ex-Vivo and In Vitro Skin Penetration Test

DMPs loaded with SRB and the letter type microneedle arrays were used to evaluate the skin penetration property. The letter type microneedle arrays fabricated in the previous study were placed in the array at intervals of 500 μm in DK letter. Before the skin penetration, the ex vivo porcine skin (CRONEX, Seoul, Korea) was thawed in water bath for 5 min. Subsequently, the fur of the skin was removed by a razor and cleaned with an alcohol swab. After drying the skin surface, DMPs and the letter-type microneedle arrays were inserted, respectively, into the skin by pressing with a thumb and then maintained for 20 min [21]. The letter type microneedle arrays were removed from the skin, and the skin was stained using 2% (*w*/*v*) gentian violet solution for 10 min. After that, the excess of gentian violet solution was cleaned with an alcohol swab. DMP, the letter type microneedle arrays, and the skin were analyzed using a stereomicroscope.

To perform in vitro skin test, the agarose gel as an artificial skin was prepared by dissolving 1% (*w*/*v*) agarose powder (LPS solution, Daejeon, Korea) in 1X PBS buffer. DMPs containing SRB were inserted into the agarose gel by pressing with a thumb for 30 s and maintained for 20 min. Then, DMPs were removed from the agarose gel, and DMP and the agarose gel were analyzed using stereomicroscope.

### 2.5. Statistical Analysis

All data are expressed as mean ± standard deviation (SD) from the data obtained at least three times. An unpaired t-test and one-way analysis of variance (ANOVA) were utilized to determine the statistical significance. In all data, a *p*-value < 0.05 was considered statistically significant.

## 3. Results

### 3.1. Optimizing the 3D Printing Condition for Microneedle Fabrication

#### 3.1.1. Printing Angle

Since a microneedle is needed to penetrate the stratum corneum and deliver drugs, it is critical to produce a high-resolution tip rather than an exact length. Moreover, the microneedles should have enough mechanical strength to penetrate the skin, which is closely related to the geometric shape of the microneedle [5,22]. We studied the 3D printing condition to fabricate microneedles with optimal tip diameter and shape. Since it is reported that pyramid-shaped microneedles have stronger mechanical strength than conical ones, the pyramidal microneedles were selected in this study [23].

**Figure 1:**
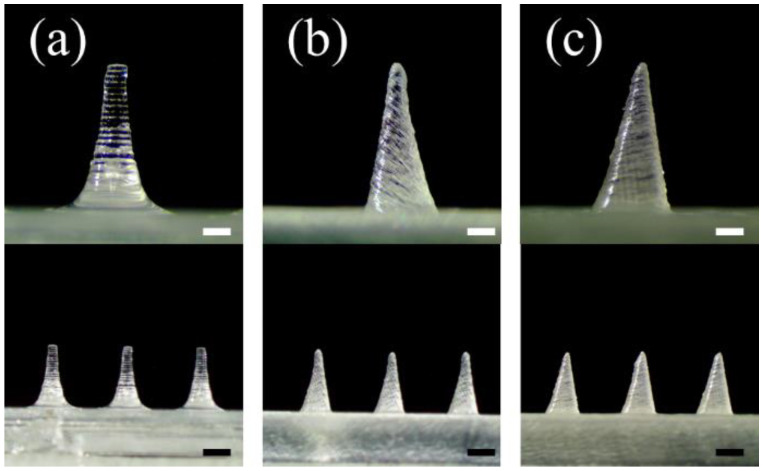
Effect of adjusting the 3D printing angle on the microneedle tip printing. Stereomicroscopic images of the microneedles at the different printing angles (**a**) 0°, (**b**) 45°, and (**c**) 60° to the x and y axes (*n* = 7; the upper and lower scale bars are 0.3 mm and 0.5 mm, respectively).

As the first parameter, the angle of 3D printing was investigated (Figure 1). The printing angle was set to 0° and then printed. A pyramid-shaped microneedle designed with 1300 µm height, and 500 µm base was printed and observed with a stereomicroscope. As a result, the base and height of the microneedle were printed similarly to the input design. However, the tip diameter was measured at 155.2 ± 1.3 µm, unlike the initially designed microneedles (Figure 1a and Table 2). Then, 3D printing was performed by changing the printing angle on one (x or y-axis) or both axes (x-y axes).

When only the x-axis was tilted at 45°, the tip diameter in the front view was measured to 92.2 ± 2.3 μm. On the other hand, the tip diameter in the side view was 131.3 ± 2.5 μm, showing a lower printing resolution (Figure S2a,c and Table S1). When only the y-axis was tilted 45°, the front view had a low resolution with a tip diameter of 131.7 ± 1.6 μm, but the side view confirmed that the tip diameter was 92.3 ± 2.6 μm, which was sharper than the front view (Figure S2b,d and Table S1). Thus, these results indicated that the printing axis should be tilted to produce a sharper microneedle tip.

Microneedles were printed by tilting both x and y axes to produce a sharp tip shape in both the front and side views (Figure 1b,c). When both axes were tilted at 45°, a sharper tip could be shown in both the front and side views, and the diameter of the tip was measured to be 92.4 ± 9.7 µm (Figure 1b and Table 2). When both axes (x-y axis) were tilted to 60°, the microneedle with the sharpest tip was printed, and the diameter of the tip was measured to be 30.2 ± 3.4 µm (Figure 1c). Therefore, it was confirmed that the control of the 3D printing angle at both x and y axes is critical for producing the sharp tip of the microneedle with the highest resolution. 

**Figure 2:**
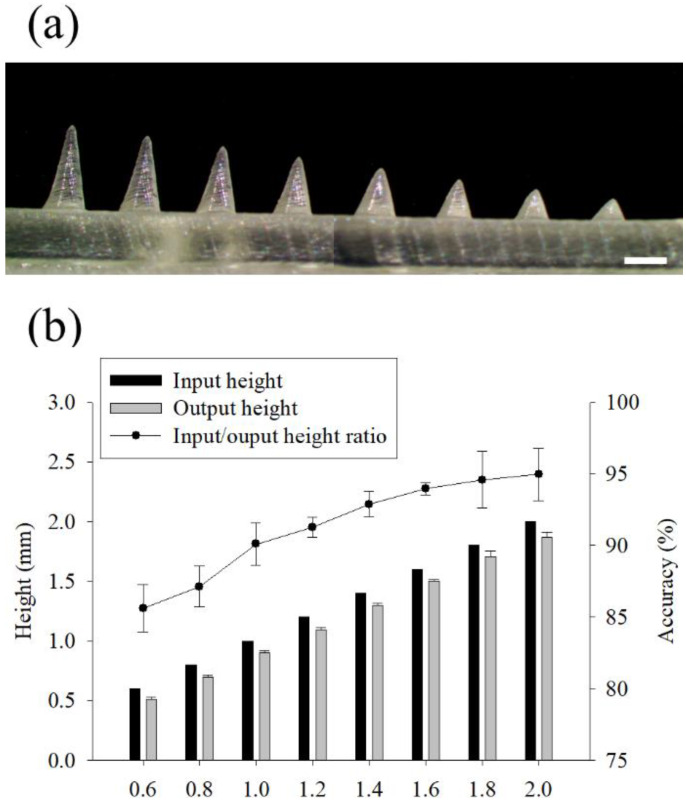
Effect of the input height of the microneedle on the 3D printing. Stereomicroscopic images of the microneedles with input height ranging from 600 to 2000 µm at 200 µm intervals (**a**). The ratio of the input to output and the 3D printing accuracy according to the microneedle height (**b**). (*n* = 7, Scale bar is 1.0 mm).

The change of the printed height of the microneedle according to the input height value was studied (Figure 2). The microneedle height was designed from 600 to 2000 µm at intervals of 200 µm, and the height of the printed microneedle was measured with a microscope (Figure 2a). The input height (computer dimension) and the output height (3D printed dimension) of the microneedle were measured, and the height difference was expressed as accuracy (%) (Figure 2b). The printed microneedle height was reduced by 101.5 ± 1.2 µm, and when the microneedle height was 600 µm, the output was reduced to at least 85.6 ± 1.7%. As the input height increased, the accuracy of the microneedle output increased, and the microneedle with an input height of 2000 µm was printed at an average of 95.0 ± 1.8%.

#### 3.1.2. Aspect Ratio and Distance between Microneedles

**Figure 3:**
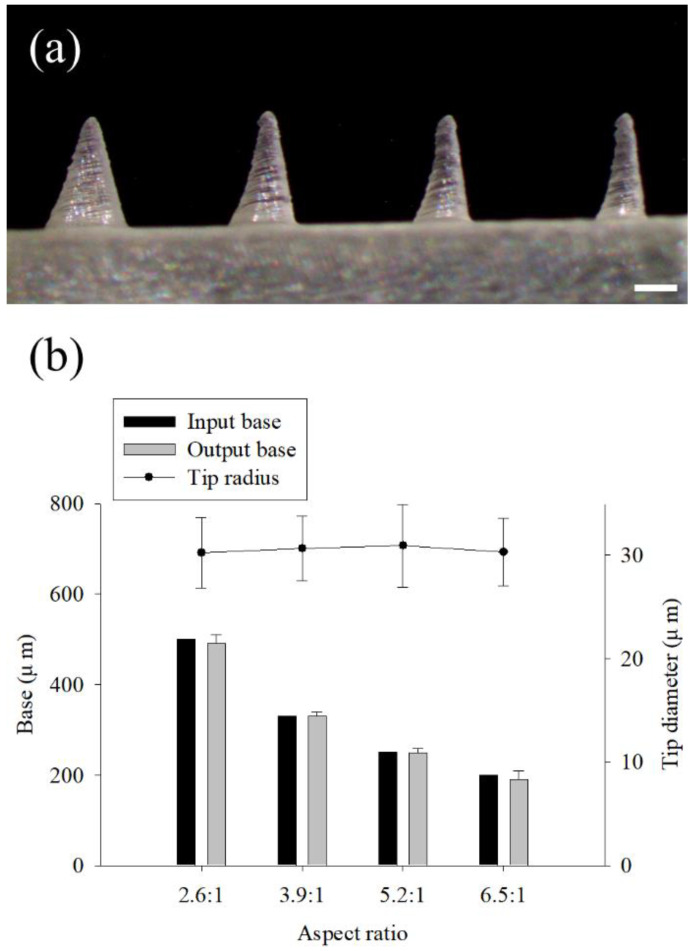
Effect of the aspect ratios of the microneedle on the 3D printing. Stereomicroscopic images of the microneedles with 1300 µm input height and 500 µm base at the aspect ratios of 2.6:1, 3.9:1, 5.2:1, and 6.5:1 (**a**). The dimensions of the input to output base and the tip diameter according to the aspect ratios (**b**) (*n* = 7, scale bar is 1.0 mm).

The 3D printing accuracy according to the aspect ratio of the microneedle was studied (Figure 3). Typically, to give sufficient mechanical strength to the microneedle, the aspect ratio is reduced or the width of the base is increased. The aspect ratio of the microneedles used for skin penetration is known to be 2:1 to 10:1 [5]. The printing angle was set to 60° for both the x and y axes, and the input height of the microneedle was fixed to 1300 µm. The base size was designed to be 500, 330, 250, and 200 µm, and the aspect ratios were 2.6:1, 3.9:1, 5.2:1, and 6.5:1, respectively (Figure 3a). The printed lengths of the microneedle base were similar in all aspect ratios when compared with the input value (computer dimension) (*t*-test, *p* > 0.88, 0.92, 0.85, and 0.74, respectively) (Figure 3b). On the other hand, the tip diameter was similar, regardless of the aspect ratio (One-way ANOVA, *p* > 0.99). Finally, the aspect ratio of 2.6:1 and the dimensions of 1300 µm in height and 500 µm in the base were utilized for further study.

The effect of the spacing between the microneedles on the 3D printing result was investigated (Figure S3). The printing angle was set to 60° with both x and y axes, and the spacing between the microneedles was designed to 1000 µm, 500 µm, and 250 µm. As a result of the 3D printing, the spacing of the microneedles did not affect the microneedle shape (Table S2). In order to avoid the “bed of nail” phenomenon and facilitate microneedle penetration into the skin, the spacing between the microneedles was designed to be 1000 µm for further research.

### 3.2. Fabrication of Dissolving Microneedle Patches (DMPs)

**Figure 4:**
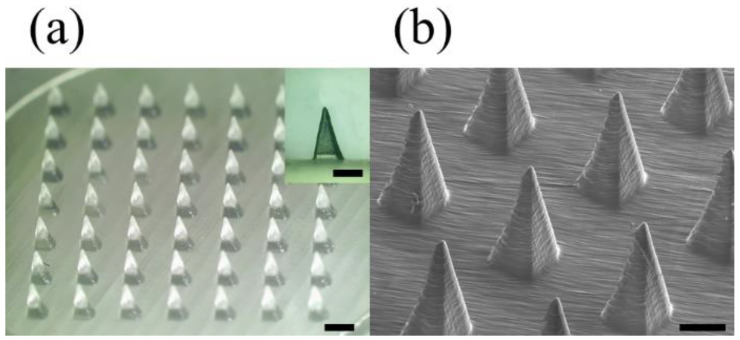
The 3D printed microneedle mold array measured by a stereomicroscope (**a**); the scale bar in the inset image and the bottom scale bar are 0.5 mm and 1.0 mm, respectively. SEM (**b**); scale bar is 0.5 mm. The microneedle mold array consisting of 7 × 7 microneedles was printed at the optimized condition with 60° printing angle to the x and y axes, 2.6:1 aspect ratio (input height 1300 µm, base 500 µm), and 1000 µm spacing between the microneedles.

A microneedle mold was printed under optimized 3D printing conditions to fabricate a dissolving microneedle patch (DMP) for skin penetration. The microneedle mold array consists of 7 × 7 microneedles, the printing angle is 60° to the x and y axes, the aspect ratio of the microneedles is 2.6:1 (input height 1300 µm, base 500 µm), and the distance between the microneedles is designed to be 1000 µm. The shape of the printed microneedle mold was observed using a stereomicroscope and SEM, and the microneedle dimensions were measured with 1180 ± 20 µm height, 490 ± 20 µm base, and 30.2 ± 3.4 µm tip diameter (Figure 4). Through the analysis of stereomicroscope and SEM images, it was confirmed that high-resolution microneedles with sharp tips were printed with consistency and reproducibility.

**Figure 5:**
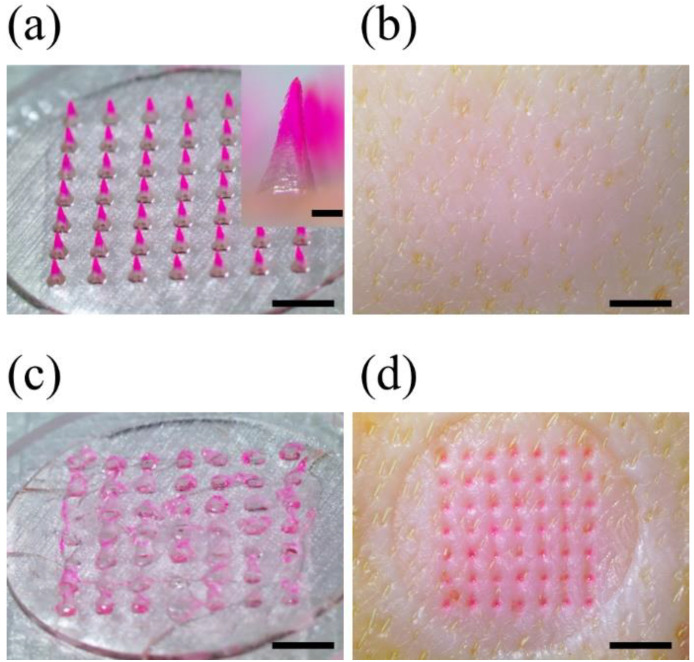
Skin penetration test of DMP into the porcine skin ex vivo. The images of DMP before (**a**) and after (**c**) the skin penetration, and the porcine skin ex vivo before (**b**) and after (**d**) the DMP skin penetration.

DMP was manufactured using the solvent casting method using 3D printed microneedle mold (Figure S1). In order to visually check whether the drug is distributed only at the microneedle tip, SRB was used as a model drug by dissolving it in the 1st cast solution; 2% (*w*/*v*) CMC-Na solution was used as the 1st cast solution, and 40% *w*/*w* PVP K-30 solution was used as the 2nd cast solution. As a result of observing the fabricated DMP under a stereoscopic microscope, it was confirmed that SRB was distributed only at the tip of the microneedle (Figure 5a). This showed that the 1st cast and the 2nd cast solution do not mix, and the model drug (SRB) is distributed only on the tip and can be efficiently delivered to the body. DMPs were measured with 1180 ± 10 μm height, 490 ± 10 μm base, and 32.4 ± 4.5 μm tip diameter, confirming that they were manufactured similarly to the microneedle mold. 

When the printing angle changes, the stacking direction changes, which weakens the mechanical strength of the microneedle mold, which may cause damage to the output. In order to prevent this, 3D printed microneedle mold was additionally cured in Form Cure equipment. As a result, it was confirmed that there was no difference in the shape of the mold and DMP, and a stable mold was fabricated (Figure 4a inset and Figure 5a inset).

### 3.3. Ex-Vivo Skin Penetration Test

To show that DMP penetrated the skin, DMP was inserted into porcine skin ex vivo for 20 min, and the porcine skin and DMP were observed before and after insertion (Figure 5a,b). All the DMP tips were dissolved and disappeared after skin penetration. Then, the porcine skin was stained with SRB as much as the microneedles, showing a 100% insertion rate (Figure 5c,d). In vitro penetration test also confirmed the microneedle penetrated the artificial skin and the agarose gel, and the surrounding area was dyed due to the released SRB (Figure S4). Therefore, the prepared DMP could penetrate the stratum corneum sufficiently and dissolve in the skin at a fast rate to be used as a suitable tool for drug delivery in the future.

### 3.4. Effect of Printing Angle on Microneedle Output Resolution

**Figure 6:**
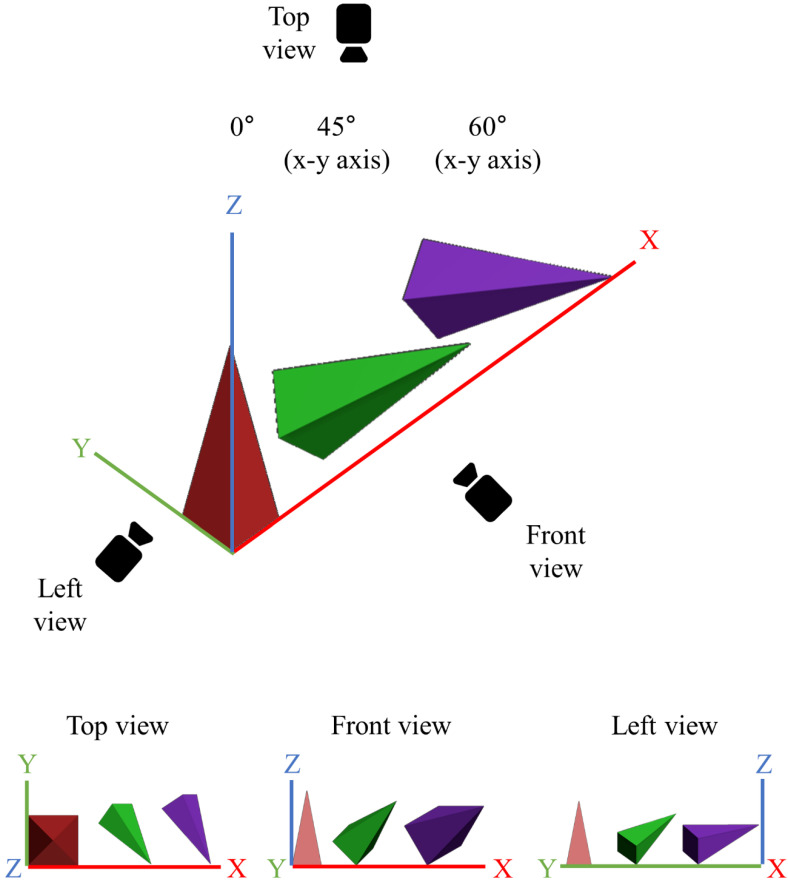
3D designs of the microneedles, which were rotated the printing angles at 0°, 45°, and 60° to both x and y axes with top, front, and left view.

In this study, we demonstrate that the printing angle improves the resolution of SLA 3D printing. Existing studies have also reported that the printing angle enhances the resolution of SLA printing. However, a detailed study on why the printing angle improves the printing resolution has not been conducted [24,25]. To investigate the effect of printing angle in detail, we first designed a pyramid-shaped microneedle in the CAD program and then tilted the 3D object according to the printing angle (Figure S5). When the printing angle was tilted only to the x-axis, as the printing angle increased from 0°, to 45°, to 60°, the microneedle was printed in a lying state, and the tip of the microneedle was also sharp only in the front view (Figure S2a). On the other hand, when both the x and y axes were tilted, a sharp tip could be seen in both the front and side views (Figure 1b,c). Moreover, as the printing angle increased from 0° to 45° and 60°, the designed microneedle was printed in a lying state (Figure 6), and the tip was also printed to be sharper (Table 2). When the printing angle was 90° (maximum printing angle), the support was created around the microneedle tip by the slicing software due to the unstable position of the microneedles (Figure S6). Since the microneedle tips were damaged when removing “the support" around the tips, they were excluded from the results in this study.

**Figure 7:**
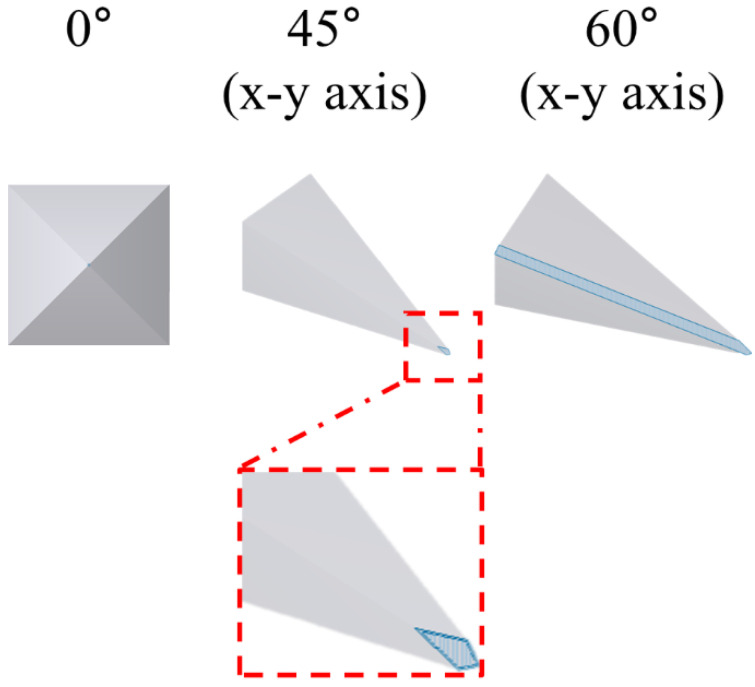
Schematic images of the single stacking layer area, including the microneedle tip at the printing angles 0°, 45°, and 60°, for both x and y axes.

The relationship between the tilted state of the microneedle and the printing resolution was verified through a slicing software (Preform, Formlabs Inc., Somerville, MA, USA). The microneedle tilted according to the printing axes, and angle was sliced from the printing stage with 25 μm thickness in the z-axis direction by the slicing program. Then, the area of the single stacking layer, including the microneedle tip, was calculated (Figure 7 and Table 3). When the microneedle was not tilted, the tip diameter was bluntly printed as 155.2 ± 1.3 μm (Figure 1a). When both the x and y axes were tilted at 45°, the area including the tip was 531 μm^2^, and the tip diameter was decreased to 92.4 ± 9.7 μm (Figure 1b). When both x and y axes were tilted to 60°, the area of the single stacking layer, including the tip, was the widest at 22,187 μm^2^, and the tip diameter was the sharpest at 30.2 ± 3.4 μm (Figure 1c). Therefore, this result indicates that when the printing angle of the microneedle is tilted correctly, a wide single stacking layer, including the tip shape, can be formed, thereby making it possible to print a high-resolution microneedle.

### 3.5. High-Dimensional Structure Microneedle Mold Printing

**Figure 8:**
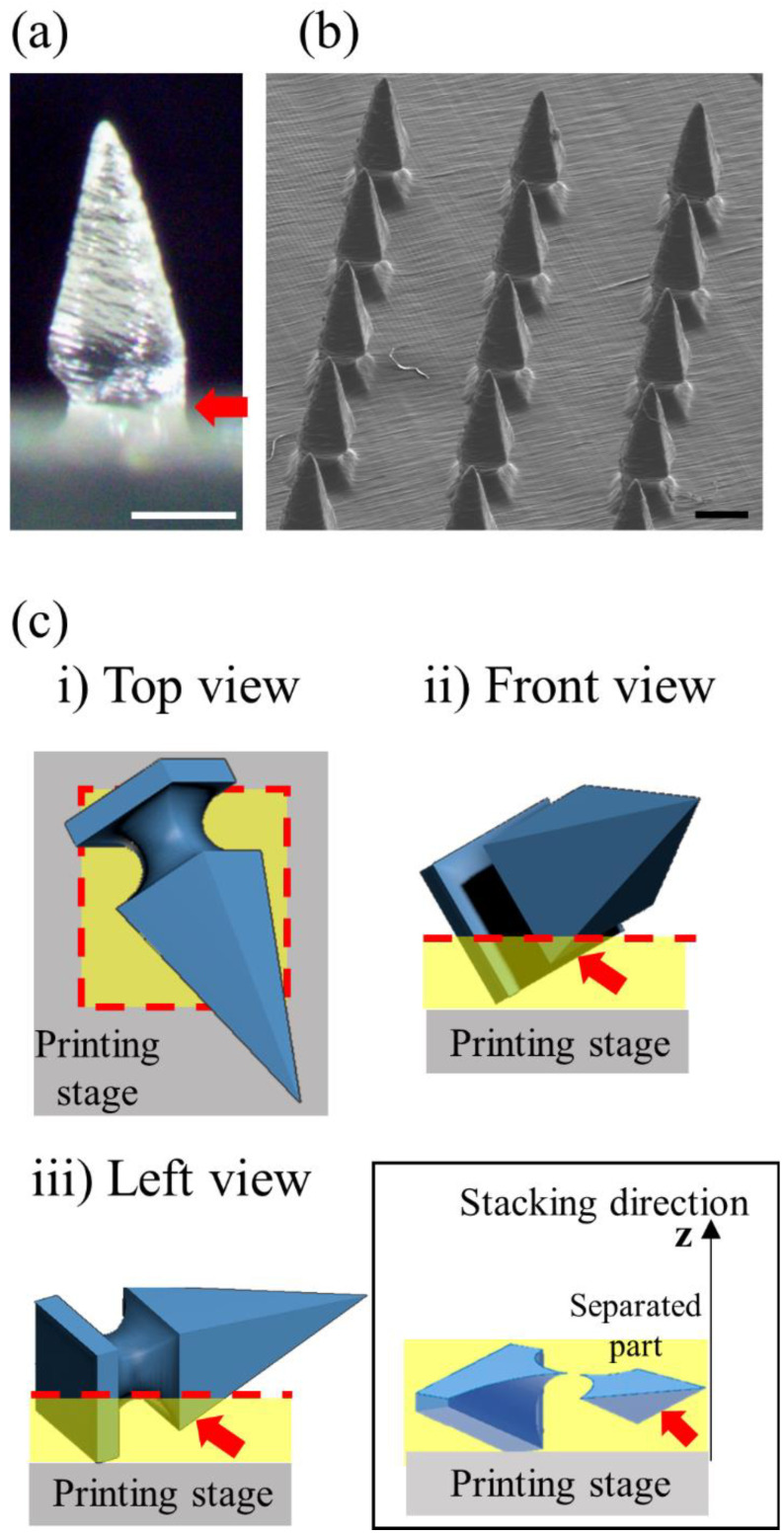
Fabrication of high-dimensional side-notched arrowhead (SNA) microneedles by optimized 3D printing. The SNA microneedles were measured using a stereomicroscope (**a**) and SEM (**b**) (All scale bars are 0.5 mm). (**c**) The 3D design of the SNA microneedles rotated to 60° printing angle for both x and y axes with (**i**) top, (**ii**) front, and (**iii**) left views. A separated part (red arrow) from the microneedle body was generated in the single stacking layer indicated by the red dotted line due to the specificity of the SNA microneedle structure (inset). The separated part was 3D printed inaccurately.

In the above study, we confirmed that the printing resolution was improved by adjusting the printing angle, thereby optimizing the SLA 3D printing condition. Based on the condition, we designed a side-notched arrowhead (SNA) microneedle mold to check whether a high-dimensional microneedle could be produced (Figure 8). Since the SNA microneedle mold has a narrow base structure, it can be used to fabricate a ‘separable microneedle patch’ where the tip parts are easily separable by shear force after the skin penetration [26]. Moreover, the separable microneedle patch can reduce the patch dwelling time for patients and control the drug-release time.

After tilting the x and y axes to 60°, the SNA microneedles were printed, and they measured the morphology with a stereomicroscope and SEM (Figure 8a,b). The arrowhead shape of the microneedle was precisely produced, but the one side of the notch (red arrow) was not accurately printed (Figure 8a). To analyze the printing results of the inaccurate parts (red arrow), the SNA microneedle design was tilted by 60° in the x and y axes, respectively, and divided the design into a single stacking layer using the slicing program (Figure 8c). The stacking direction of the printing proceeds to the z-axis direction from the printing stage. When the single stacking layers of the SNA microneedles were analyzed, it was found that a part of the SNA microneedle (red arrow) was separated from the microneedle body and printed (Figure 8c inset). Since this separated part has no supporting part under it, the part falls off from the microneedle even if it is printed. Therefore, it is confirmed that one side of the notch is challenging to print correctly. However, the entire SNA microneedle array and tip structure were accurately printed, and the notches on the remaining sides were printed (Figure 8b). Consequently, this study proved that high-dimensional microstructures can be printed with an improved resolution by varying the printing angle.

### 3.6. Printing the Microneedles of the Letter Type Array

**Figure 9:**
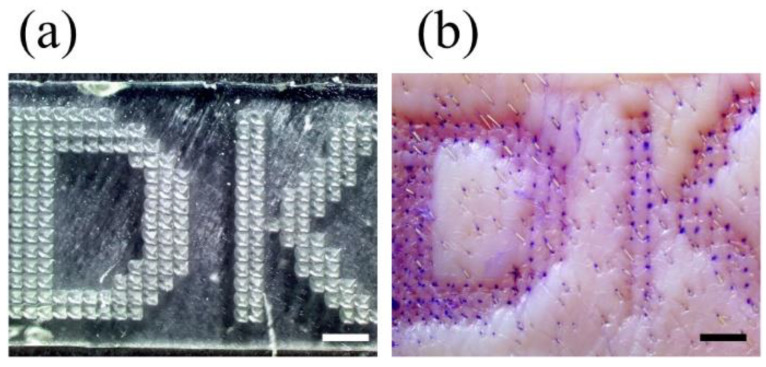
The letter type microneedle arrays were measured by a stereomicroscope (**a**). The skin penetration image of the array into the porcine skin ex vivo (**b**) (scale bars are 2.0 mm).

One of the 3D printing advantages is that it can print 3D objects in a customized arrangement [27]. To show that custom high-resolution arrays can be easily manufactured using an SLA 3D printer, we fabricated a letter type microneedle array (Figure 9). The skin penetration was confirmed by inserting a microneedle array in a DK letter into the porcine skin ex vivo (Figure 9a). As a result, the skin was stained according to the letter shape, and the skin penetration rate was about 98% (Figure 9b). Therefore, we proved through this study that the microneedle produced by 3D printing can be arranged in various ways to be applied not only for drug delivery but also to tattoo, text, barcode, or QR codes.

## 4. Discussion

SLA 3D printing is a 3D printing method with high resolution and can easily design and print microneedles using a CAD program. Microneedle fabrication using an SLA-type 3D printer has been reported in various studies [28,29,30]. However, the limitation of microneedle fabrication using 3D printing was that the microneedle tip, which is essential for skin penetration, could not be accurately produced because of the low resolution of 3D printing. When microneedles are designed with a CAD program and 3D printed, generally, the backing layer and base part of microneedles are produced almost identically to the input design due to a sufficiently wide single stacking layer (with no printing angle). On the other hand, the closer one gets to the microneedle tip, the narrower the single stacking area; the limit that cannot be produced with 3D printing is reached, and the tip shape is printed inaccurately. In this study, we hypothesized that if the stacking area, including the tip, is widened, the microneedle tip shape can be sufficiently printed even with a low-resolution 3D printer. When the 3D printing angle was adjusted, the stacking area, including the microneedle tip, increased as the stacking direction of the object was changed (Figure 7). Therefore, we could fabricate high-resolution microneedles by adjusting the printing angle.

3D printing studies for improving resolution to fabricate microscale objects have been reported, Hada et al. We investigated the 3D printing conditions in which the printing result was almost identical to the input design when the printing angle was changed to 0°, 45°, and 90° [24]. The surface area of the printed object was checked using a 3D optical scanner and compared with the design. The case with the lowest difference between the input and output was reported as the optimal condition, and it was confirmed that the most accurate output was obtained when the printing angle was 45°. However, this study did not investigate the cause of why the resolution is the best when the printing angle is 45°. Yeung et al. manufactured a hollow microneedle for transdermal drug delivery using a 3D printer [31]. In this paper, the microneedle was printed by changing the printing angle to 0°, 45°, and 90°, respectively. The author revealed that excellent microneedle could be printed when the printing angle was set to 45° due to the resin flow but did not report the cause of the improved resolution.

Balmert et al. fabricated microneedles of various shapes with high dimensional precision at the level of several hundred nanometers using a 2PP-type 3D printer [32]. However, 2PP-type 3D printing takes a long time, and the equipment is expensive. Jun et al. manufactured the microneedle tip and the base separately [33]. The author produced microneedles where the tip can be easily separated from the base when the microneedle penetrates the skin. For easy separation of the tip, a small 100 μm high wall was made on one side of the base through photolithography. Although the previous studies produced sophisticated high-dimensional microneedles, the fabrication process is complicated, time-consuming, and needs expensive equipment. Furthermore, it is difficult to revise and supplement the design of the microneedles. However, the 3D printing method we presented in this study has the advantage of fabricating a high-dimensional microstructure rapidly with one step by adjusting the printing angle. Moreover, if the design needs to be revised, it can be easily modified with a CAD program, and the result can be printed immediately. Therefore, the study we presented will be usefully applied to inexpensively and rapidly fabricating high-dimensional microstructures. 

## 5. Conclusions

In this study, we demonstrated that high-resolution and high-dimensional microneedles were manufactured by adjusting the printing angle of the SLA-type 3D printer. When the printing angle was set to 60° for the x and y axes, the single stacking layer area, including the microneedle tip, was the largest, and the microneedle with the sharpest tip was printed. The 3D printed microneedle under optimal conditions was manufactured as a dissolving microneedle using the solvent casting method, and it had mechanical strength capable of penetrating the skin. Furthermore, a side-notched arrowhead (SNA) microneedle with a high-dimensional structure could be fabricated by adjusting the 3D printing angle. Therefore, we presented a method for manufacturing microneedles quickly and simply without the need for expensive facilities and equipment, confirming the 3D printing technology could be used in various fields and drug delivery.

## Figures and Tables

**Table 2:** Dimensions of the printed microneedles at different 3D printing angles 0°, 45°, and 60° to the x and y axes (*n* = 7).

Printing Angle of x-y Axes	Design	0°	45° (x-y Axes)	60° (x-y Axes)
Tip diameter (μm)	1 *	155.2 ± 1.3	92.4 ± 9.7	30.2 ± 3.4
Height (μm)	1300	1280 ± 10	1230 ± 10	1200 ± 10
Base (μm)	500	490 ± 10	490 ± 20	490 ± 20

* We assumed the tip diameter of the pyramid microneedle design is 1 μm.

**Table 3:** The area of the single stacking layers, including the microneedle tip at the printing angles 0°, 45°, and 60°, for both x and y axes.

Printing Angle of x-y Axes	0°	45° (x-y Axes)	60° (x-y Axes)
Area (μm^2^)	0.8 *	531	22,187

* We assume that the single stacking area is a circle with a 1 μm diameter.

## Data Availability

Not applicable.

## Supplementary Materials

The following supporting information can be downloaded at: https://www.mdpi.com/article/10.3390/pharmaceutics14040766/s1, Figure S1: Schematic illustrating the fabrication of a dissolving microneedle patch (DMP); Figure S2: Effect of the axis direction and the printing angle on the microneedle tip printing; Table S1: Dimension of the microneedle tip diameter 3D printed at the x or y single-axis with 45° printing angle; Figure S3: Effect of the spacing between the microneedles on the microneedle tip printing; Table S2: Dimensions of the printed microneedles at different spacing between the microneedles 1000 µm, 500 µm, and 250 µm; Figure S4: Penetration test of DMP into the agarose gel in vitro; Figure S5: 3D designs of the microneedles, which were rotated the printing angles at 0°, 45°, and 60° to the x-axis; Figure S6: 3D designs of the microneedles, which were rotated the printing angles at 90°; Table S1: Dimension of the microneedle tip diameter 3d printed at the x or y single-axis with 45° printing angle (*n* = 7); Table S2: Dimensions of the printed microneedles at different spacing between the microneedles 1000 µm, 500 µm, and 250 µm (*n* = 7).
