# Supplementary material for: Fabricating High-Resolution and High-Dimensional Microneedle Mold through the Resolution Improvement of Stereolithography 3D Printing
Source: Pharmaceutics. 2022 Mar 31;14(4):766. doi: 10.3390/pharmaceutics14040766 (PMC9027855; doi:10.3390/pharmaceutics14040766)
Supplement: Supplementary file 1 [file pharmaceutics-14-00766-s001.zip › pharmaceutics-1648604-supplementary.pdf]

pharmaceutics

Supplementary Materials

Fabricating High-Resolution and High-Dimensional Microneedle Mold Through the Resolution Improvement of Stereolithography 3D Printing

Sangmin Choo, Sung Giu Jin * and Jae Hwan Jung *

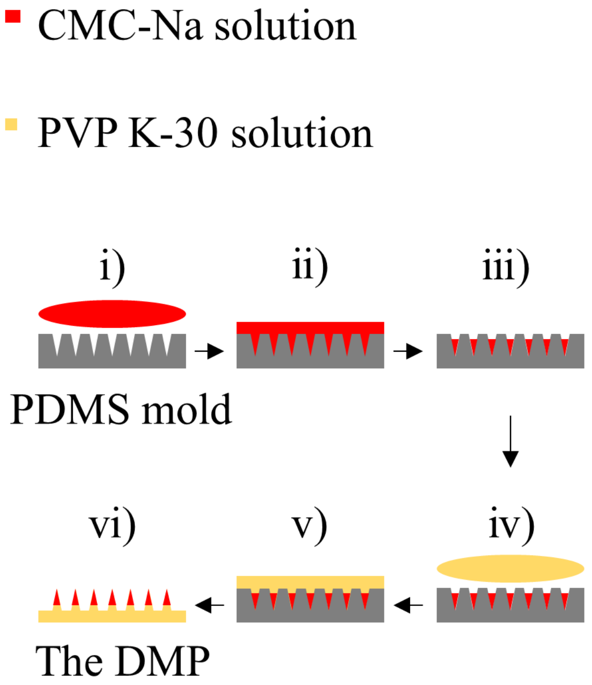

Figure S1. Schematic illustrating the fabrication of a dissolving microneedle patch (DMP). (**i**) Casting of 1st cast solution. (**ii**) Vacuum for 1 h. (**iii**) Remove excess solution and dry for 1 h. (**iv**) Casting of 2nd cast solution. (**v**) Vacuum for 1 h. (**vi**) Dry for 24 h at 25 °C and peel off from PDMS mold.

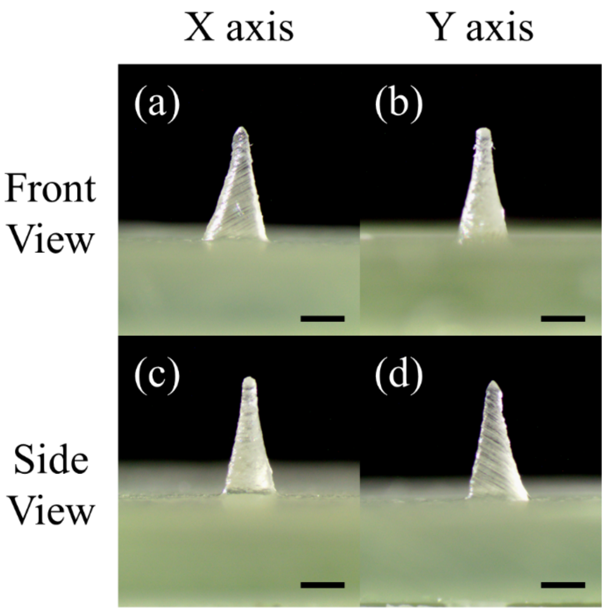

Figure S2. Effect of the axis direction and the printing angle on the microneedle tip printing. The microneedle images 3d printed at 45° printing angle to the x-axis: front view (**a**) and side view (**c**); at 45° printing angle to the y-axis: front view (**b**) and side view (**d**) (scale bar is 0.5 mm).

Table S1. Dimension of the microneedle tip diameter 3d printed at the x or y single-axis with 45 ° printing angle (n = 7).

Printing Degree of x-y Axis	45° (x Axis) (Front View)	45° (x Axis) (Side View)	45° (y Axis) (Front View)	45° (y Axis) (Side View)
Tip diameter (μm)	92.2 ± 2.3	131.3 ± 2.5	131.7 ± 1.6	92.3 ± 2.6

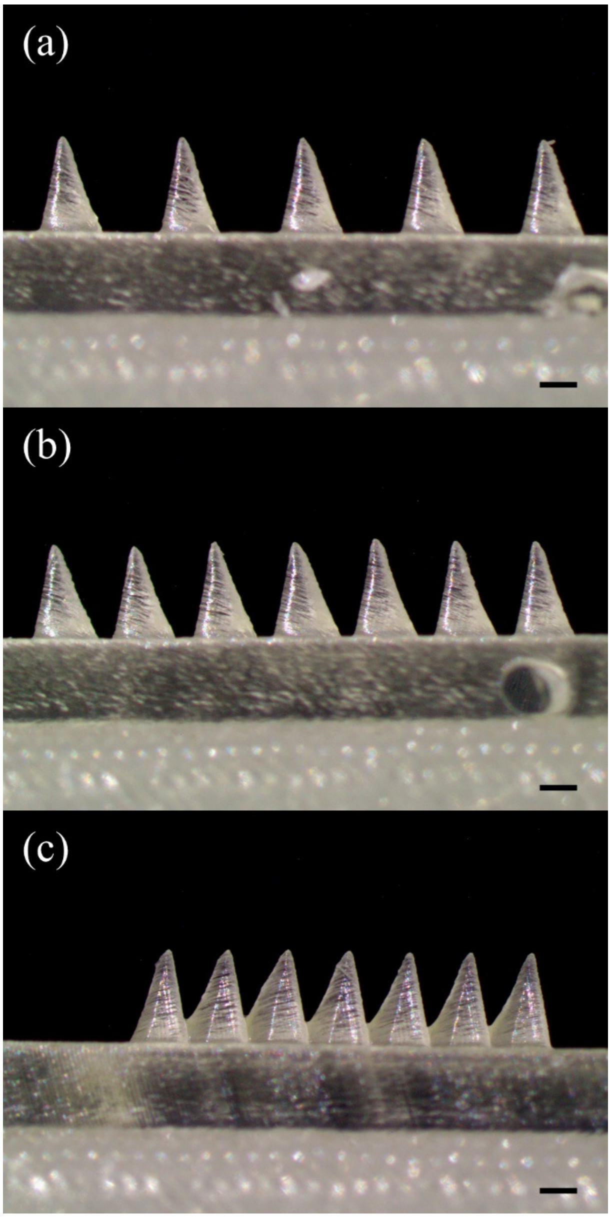

Figure S3. Effect of the spacing between the microneedles on the microneedle tip printing. Stereomicroscopic images of the microneedles (1300 μm input height and 500 μm base) at the spacing of 1000 μm (**a**), 500 μm (**b**), and 250 μm (**c**). (n = 7, scale bar is 0.5 mm).

Table S2. Dimensions of the printed microneedles at different spacing between the microneedles 1000 μm, 500 μm, and 250 μm (n = 7).

Spacing between the Microneedles	Design	1000 μm	500 μm	250 μm
Tip diameter (μm)		30.2 ± 3.4	30.3 ± 3.5	30.4 ± 4.5
Height (μm)	1300	1200 ± 10	1200 ± 20	1210 ± 20
Base (μm)	500	490 ± 20	490 ± 10	490 ± 10

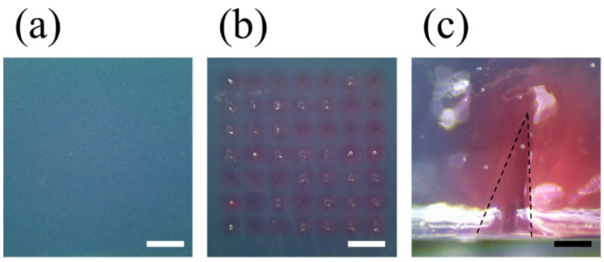

Figure S4. Penetration test of DMP into the agarose gel in vitro. Images of the agarose gel before (**a**) and after (**b**), and cross-section of the agarose gel (**c**) after the agarose gel penetration test (the white scale bars and black scale bar are 2.0 mm and 0.5 mm, respectively).

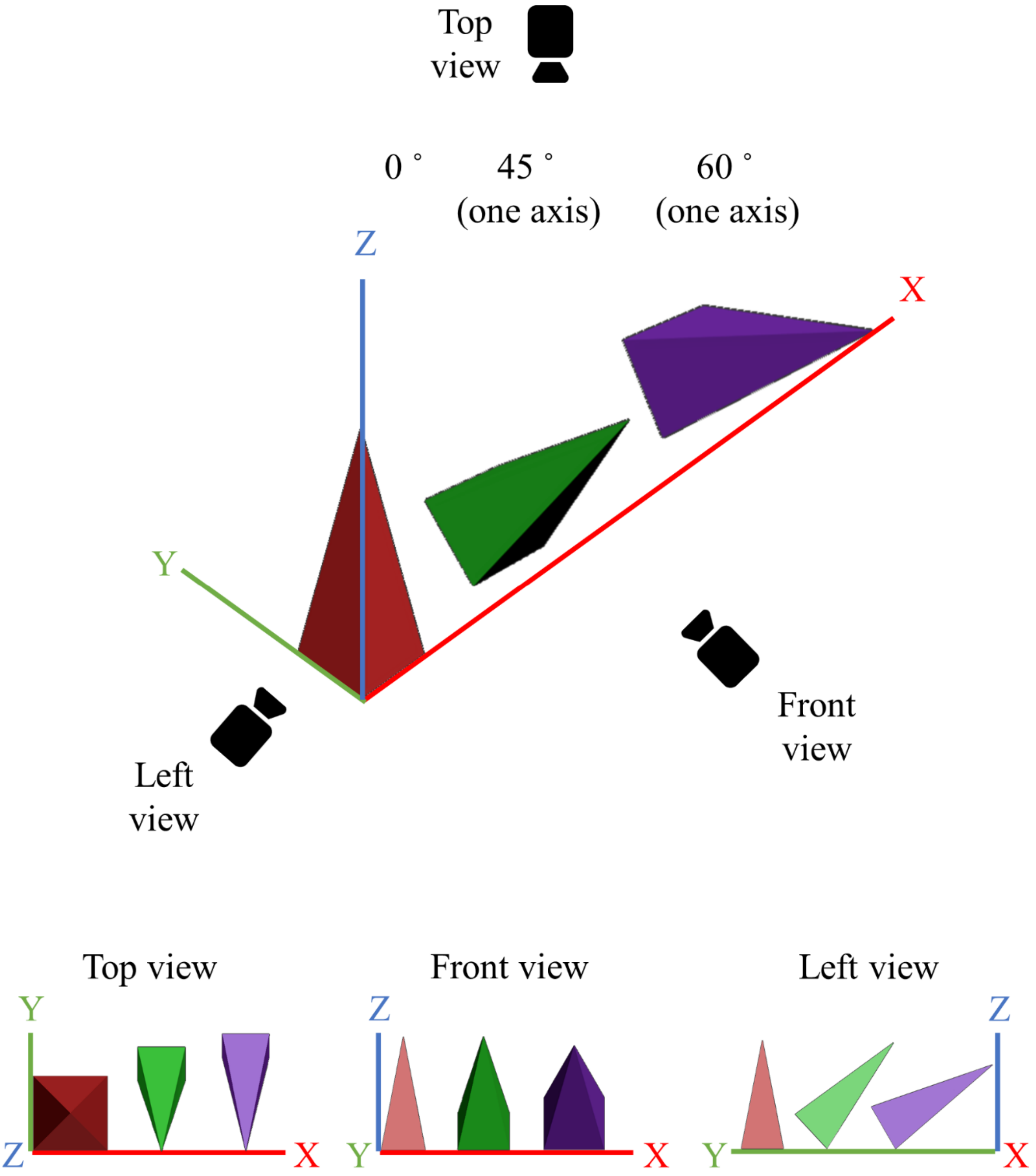

Figure S5. 3D designs of the microneedles, which were rotated at printing angles of 0°, 45°, and 60° relative to the x-axis.

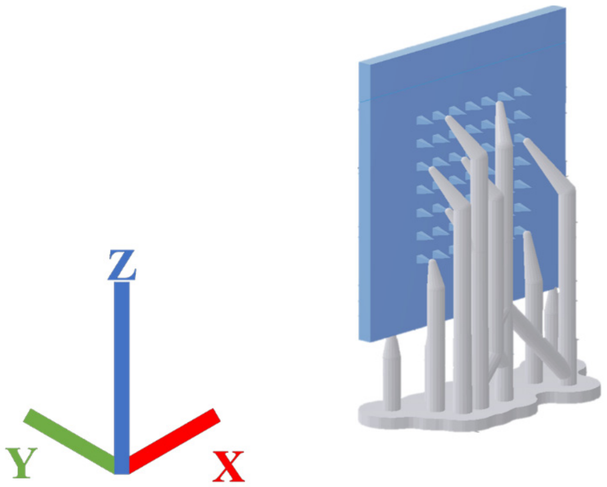

Figure S6. 3D designs of the microneedles, which were rotated at a printing angle of 90°. Supports around the microneedle tips were generated due to the unstable position of the microneedles at a 90° printing angle.
